# Characterization of Fatigue Crack Growth Based on Acoustic Emission Multi-Parameter Analysis

**DOI:** 10.3390/ma15196665

**Published:** 2022-09-26

**Authors:** Mengyu Chai, Chuanjing Lai, Wei Xu, Quan Duan, Zaoxiao Zhang, Yan Song

**Affiliations:** 1 School of Chemical Engineering and Technology, Xi’an Jiaotong University, Xi’an 710049, China; 2 BASF Chemicals Company Ltd., Shanghai 201507, China

**Keywords:** acoustic emission, fatigue crack growth, multi-parameter, online monitoring, damage characterization

## Abstract

In engineering structures that are subject to cyclic loading, monitoring and assessing fatigue crack growth (FCG) plays a crucial role in ensuring reliability. In this study, the acoustic emission (AE) technique was used to monitor the FCG behavior of 2.25Cr1Mo0.25V steel in real-time. Specifically, an AE multi-parameter analysis was conducted to qualitatively assess the crack growth condition and quantitatively correlate the crack growth rate with AE. Various AE parameters were extracted from AE signals, and the performances of different AE parameters were analyzed and discussed. The results demonstrated that four stages of FCG, which correspond to macrocrack initiation, stable crack growth with low crack growth rate, stable crack growth with high crack growth rate, and unstable crack growth, are distinctly identified by several AE time domain parameters. The sudden and continuous occurrence of many AE signals with high count (>100) and high energy (>40 mV·ms) can provide early and effective warning signs for accelerated crack growth before final failure occurs. Moreover, linear correlations between crack growth rate and different AE parameters are established for quantifying crack growth. Based on the AE multi-parameter analysis, it was found that the count, energy, and kurtosis are superior AE parameters for both qualitatively and quantitatively characterizing the FCG in 2.25Cr1Mo0.25V steel. Results from this research provide an AE strategy based on multi-parameter analysis for effective monitoring and assessment of FCG in engineering materials.

## 1. Introduction

As a valuable structural health monitoring technique, the acoustic emission (AE) technique has been widely used for the real-time damage monitoring and integrity assessment of materials and structures in a variety of situations. Acoustic emissions are essentially the transient stress waves that are produced as a result of the rapid release of energy from localized sources within material under stress [1]. The primary AE sources include plastic deformation, initiation and growth of cracks, fracture, corrosion, fiber debonding and pull-out, delamination in composites, and so forth [1,2]. By using a high-sensitivity piezoelectric AE sensor, an amplifier, and an advanced data acquisition system, the stress waves generated by these active sources can be detected, amplified, and converted to a digital signal. Abundant information including the time domain parameters such as amplitude, energy, count, root mean square (RMS), and so on, and frequency domain parameters such as centroid frequency can be further extracted from the collected AE signals for characterizing the damage condition of the material.

Several mechanical structures, such as pressure vessels, bridges and rails, are subject to cyclic loading that can initiate and grow fatigue cracks. For the safety and reliability of these structures, it is imperative to monitor the progression of fatigue cracks to provide early warning signs before fatigue failure occurs. Some nondestructive testing (NDT) methods such as infrared thermography (IRT) [3] and digital image correlation (DIC) [4] have been developed for detecting fatigue cracks. However, these techniques are essentially surface measurements and are only useful for the detection of surface or sub-surface damages. One of the advantages of the AE technique over these NDT methods is that it can reveal information regarding the damage development inside the material by measuring stress waves using a proper sensor. Up to now, there have been numerous investigations conducted using the AE technique to monitor fatigue processes and characterize the crack growth behavior in various materials. In particular, several methods based on AE parameters have been proposed to detect different stages of fatigue crack growth (FCG) [5,6,7,8,9,10,11]. For example, the AE behaviors of fatigue crack propagation in low-alloyed steel and its welds were studied by Han et al. [5]. By calculating the normalized AE counts as a function of fatigue cycles, three distinct stages during the fatigue process were observed. Li et al. [6] studied the curves of AE count rate (*dC*/*dN*) as a function of stress intensity factor range (Δ*K*) in commercial-purity zirconium and divided the AE into three stages, which corresponded to the three common stages of the curve of fatigue crack growth rate (da/dN) as a function of Δ*K*. The study by Aggelis et al. [7] presented a method using the rise angle (RA) of AE waveforms to characterize the damage accumulation and the change in fracture mode in the metal plate. Specifically, the RA value underwent a sharp increase prior to the final fracture, indicating the shift from the tensile to the shear fracture modes. Work by Yu et al. [8] recommended the use of AE absolute energy for characterizing FCG and quantifying crack length instead of AE count in steel bridge components because the absolute energy is less dependent on threshold-setting. In more recent studies, the AE information entropy was proposed as an effective parameter for detecting critical damage and recognizing different stages of crack growth [9,10,11]. For instance, Karimian et al. [11] calculated information entropy from AE waveforms generated in the fatigue process in aluminum alloy for detecting the point of crack initiation. The results demonstrated that, in comparison with traditional AE parameters such as count and energy, the information entropy was capable of providing a more accurate and earlier identification of fatigue crack initiation because it is threshold independent and less susceptible to attenuation.

Note that the above investigations mainly focus on the qualitative characterization of FCG with the aid of AE. Moreover, the effort has also been made to quantitatively correlate multiple AE parameters with da/dN or Δ*K* for predicting the fatigue crack length [12,13,14,15,16,17,18]. It is widely known that, during FCG, the crack growth rate can be described by the classical Paris–Erdogan equation [19]:

(1) log(da/dN)=mlogΔK+logC

where *m* and *C* are material constants. Roberts et al. [12,13] found there was an approximately linear relationship between AE count rate log(dη/dN) and logΔ*K* of S275 JR steel plate during FCG, which can be represented by a formula similar to the Paris–Erdogan law. This formula can be expressed by:

(2) log(dη/dN)=plogΔK+logB

where *p* and *B* are material constants and can be determined by experiments. By eliminating logΔ*K* from Equations (1) and (2), the relationship between log(da/dN) and log(dη/dN) can be obtained:

(3) $$\log(da/dN)=\frac{m}{p}\log(d\eta /dN)+\log C-\frac{m}{p}\log B$$

Similarly, Li et al. [14] and Keshtgar et al. [15] also proposed a linear relationship between log(da/dN) and log(dη/dN) during FCG processes of rail steel and aluminum alloy, respectively, and performed the prediction of fatigue crack size accordingly. In addition to the AE count rate, some researchers established similar relationships between log(da/dN) and other AE parameters such as energy rate [16], duration rate [17] and information entropy rate [18] for predicting the crack growth in various materials.

However, despite the fruitful research findings aforementioned on the characterization of FCG, these studies are limited to analyzing one or few AE parameters, and they rarely compare a sufficient number of AE parameters with different physical meanings. This is important because the use of improper AE parameters may lead to difficulties in characterizing FCG behavior such as the inaccurate detection of fatigue cracks or an unfavorable quantitative relationship between AE and crack growth rate. For example, previous studies [12,17] have reported that, in some specific experiments, there are significant fluctuations in the plots of AE parameters (for example, AE count rate) as a function of da/dN or Δ*K* in double logarithmic coordinates, which is not helpful for predicting the crack growth rate using AE data. To achieve more excellent performance, an AE multi-parameter analysis needs to be performed to examine the viability of different parameters and determine the superior parameters for characterizing FCG. The analysis of multi-parameter is capable of reducing the uncertainties in using only a single or limited AE parameters and has been successfully applied in damage assessment of various materials such as metal, fiber reinforced plastics and sandstone [18,20,21,22]. Therefore, in the present research work, an AE multi-parameter analysis was conducted to qualitatively assess the crack growth condition and quantify the fatigue crack growth rate with AE. A variety of AE parameters were analyzed and evaluated using time domain and frequency domain parameters extracted from AE original waveforms obtained from the FCG process of CrMoV steel.

## 2. Material and Methods

### 2.1. Fatigue Crack Growth Test

**Figure 1:**
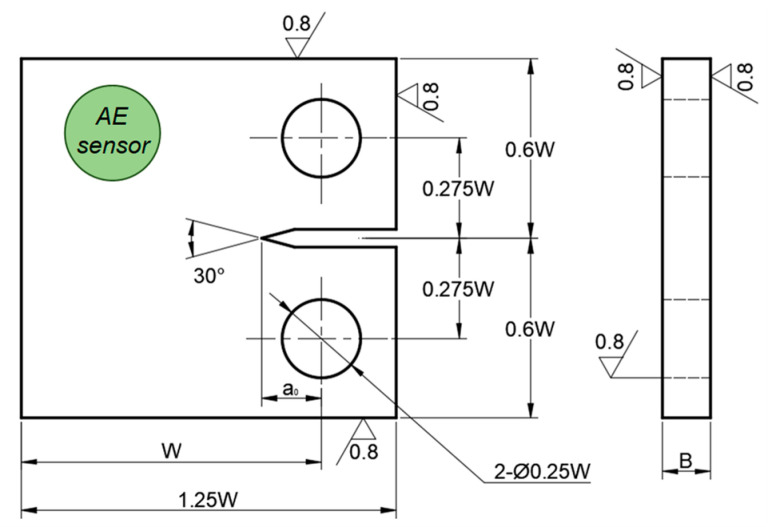
The schematic illustration of the CT specimen and position of the AE sensor.

The testing material investigated in the present work is 2.25Cr1Mo0.25V steel, which is a high strength low alloy steel and commonly used in fabrication of pressure vessels and reactors in the petrochemical industry. The main chemical composition (in wt.%) of the CrMoV steel was 0.15 C, 0.10 Si, 2.30 Cr, 0.98 Mo, 0.30 V, 0.54 Mn, 0.05 Al and balance Fe. The compact tension (CT) specimen was machined from a CrMoV steel plate to perform the FCG test at room temperature. The design of the specimen size (see Figure 1) is based on the ASTM E647 standard [23]. Specifically, the width (W) of the specimen is 55 mm and the thickness (B) is 12.7 mm (0.5 inches). The fatigue test to obtain the full crack growth curve was conducted on a servo-hydraulic testing machine (Instron, Norwood, MA, USA) following the experimental procedure in ASTM E647 standard. The specimen was subjected to a sinusoidal cyclic load with a maximum peak load of 26 kN, a load ratio of 0.5, and a loading frequency of 15 Hz to facilitate the crack growth from the notch. To measure the crack size increment during fatigue loading, the direct current potential drop (DCPD) method was used because of its advantages of high-resolution and good measurement stability. After the fatigue test, a monotonic tension load with a constant displacement rate of 2 mm/min was further applied to the specimen until complete fracture. Two tests were performed to ensure the data consistency. The fracture surface morphology was observed for clarifying the fatigue mechanism using a field emission scanning electron microscope (FE-SEM, MAIA3LMH, TESCAN, Brno, Czech Republic).

### 2.2. AE Monitoring Instrument

The AE signals generated during the FCG process were collected by using an advanced AE monitoring system (Physical Acoustic Corporation, West Windsor Township, NJ, USA) including a high-sensitivity AE sensor, a preamplifier, and a data acquisition system. The AE sensor, R15α, is a resonant piezoelectric sensor that has a resonant frequency of 150 kHz and an operating frequency range of 50–400 kHz. The sensor was fixed on the specimen surface using vacuum silicone grease as couplant and the position of the sensor is shown in Figure 1. The preamplifier gain of 40 dB was used for amplifying the AE waveform, and the data acquisition system finally converted the waveforms to digital signals for further storage and analysis. The AE system was checked for proper operation prior to the fatigue test using pencil lead break tests at multiple positions on the specimen surface. The AE monitoring was immediately performed after the onset of fatigue loading and was stopped until final fracture occurred. During the test, a sampling rate of 1 MHz was used to record AE signals, with a total sample length of 4096 points per AE event. For the small-scale specimen in the present research work, the time-driven parameters in the AE system, such as peak definite time, hit definite time, and hit lookout time, were set to 300, 600, and 1000 μs, respectively. Moreover, an analog band-pass filter of 100–400 kHz was employed to reduce the environmental and mechanical noises. Multiple parameters such as amplitude, count, entropy, RMS, and so forth, were extracted from AE signals for characterizing the FCG behavior. The extraction of these AE parameters will be presented in the next section.

### 2.3. Extraction of Multiple AE Parameters

To perform the AE multi-parameter analysis, eight time domain parameters including amplitude, count, energy, information entropy, rise angle (RA), root mean square (RMS), kurtosis and crest factor were first extracted from each AE signal recorded during the fatigue process for characterizing the FCG behavior. Furthermore, the Fast Fourier Transform (FFT) was performed to provide frequency information about AE signals because the frequency is generally regarded as an effective measure in discriminating different failure types [24,25]. Thus, in this study, the centroid frequency was calculated from the frequency spectrum to investigate the change in the frequency of AE signals during FCG. The definitions of these parameters can be found in several research journal papers and books [1,9,20,26]. The explanations of some important AE parameters used in this study are presented in Table 1.

**Figure 2:**
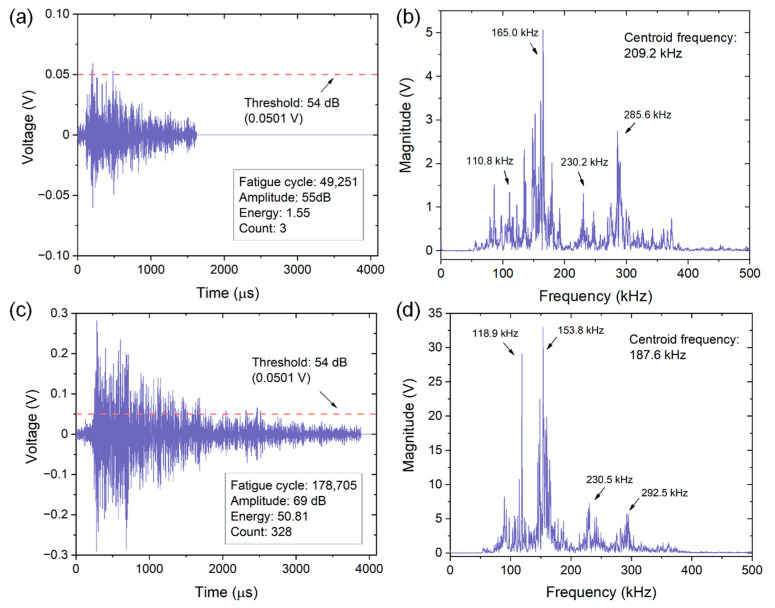
Time domain waveforms (**a**,**c**) and their corresponding FFT spectrums (**b**,**d**) of AE signals detected at the fatigue loading cycle of (**a**,**b**) 49,251 and (**c**,**d**) 178,705 of specimen No. 1. The threshold is also included in the time domain waveform for comparing the peak amplitude of two signals.

Figure 2 shows how different AE parameters are extracted from both time domain and frequency domain waveforms. Two AE waveforms collected at different fatigue loading cycles and corresponding FFT spectrum are presented. Specifically, the waveform in Figure 2a was captured at the fatigue cycle of 49,251 corresponding to the initial stage of crack growth. Obviously, the peak amplitude of this signal is 55 dB, which just exceeds the preset threshold. A small energy of 1.55 and a low count of 3 are therefore obtained because these two parameters are related to the threshold. On the other hand, the waveform in Figure 2c collected near the end of the crack growth test (178,705 cycles) exhibits an evidently larger amplitude of 69 dB, which is significantly higher than the threshold. Consequently, a larger energy of 50.81 and a higher count of 328 are obtained. By comparing the FFT spectrums of two signals, as shown in Figure 2b,d, quite similar frequency peaks and centroid frequency can be found, indicating the almost same failure type.

## 3. Results and Discussion

### 3.1. Fatigue Crack Growth Behavior

**Figure 3:**
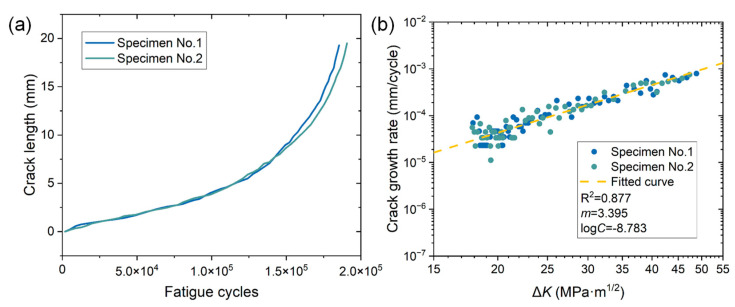
(**a**) Fatigue crack length and crack growth rate versus fatigue cycles of 2.25Cr1Mo0.25V steel. (**b**) Fatigue crack growth rate versus stress intensity factor range (Δ*K*) in the double logarithm coordinate.

Figure 3a shows the variations of fatigue crack length as a function of fatigue cycle *N* of two specimens. The final crack sizes are close to 20 mm and the fatigue lives reach approximately 190,000 cycles. The crack growth rate is calculated based on the secant method, which can be expressed as follows:

(4) $$da/dN=(a_{i+1}-a_{i})/(N_{i+1}-N_{i})$$

where da/dN is the crack growth rate, $a_{i}$ is the *i*th fatigue crack size, and $N_{i}$ is the i-th fatigue cycle. The computed da/dN is an average rate defined by the ratio of the crack size increment $\left(a_{i+1}-a_{i}\right)$ to the fatigue cycles increment $\left(N_{i+1}-N_{i}\right)$. Figure 3b exhibits the variations of da/dN as a function of Δ*K* in double logarithmic coordinates for both specimens. The da/dN almost linearly increases with Δ*K* and the data points can be fitted using the classical Paris–Erdogan crack growth law. The Paris–Erdogan law constants and the fitting coefficient R^2^ can be found in Figure 3b. The computed crack growth rate behavior will be further correlated with the AE multi-parameter analysis for characterizing different stages of FCG, which will be presented in the next section.

### 3.2. Characterization of FCG via AE Multi-Parameter Analysis

#### 3.2.1. AE Time Domain Parameters

**Figure 4:**
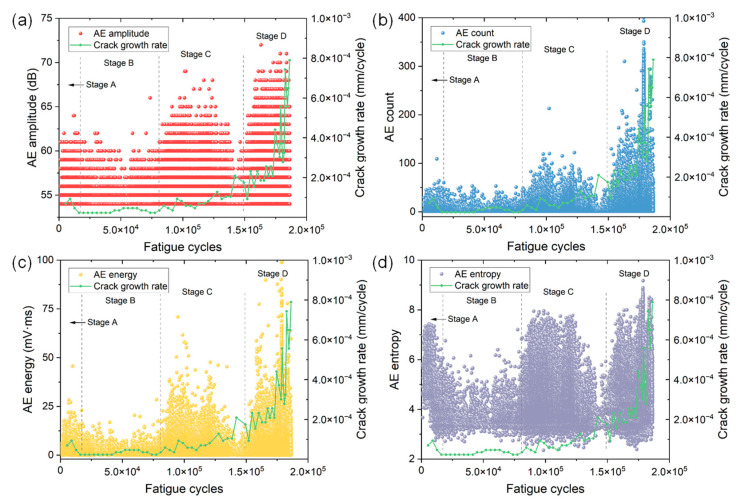
The variations of (**a**) amplitude, (**b**) count, (**c**) energy and (**d**) entropy versus fatigue cycles of specimen No. 1. The change in crack growth rate is also included for understanding the AE behaviors in different stages.

**Figure 5:**
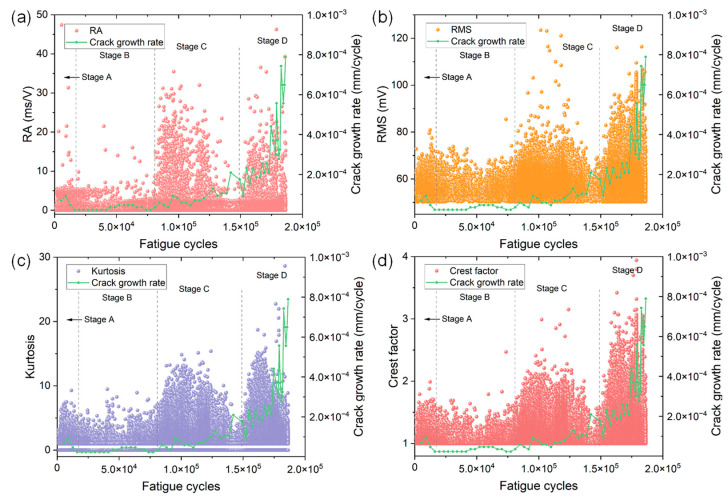
The variations of (**a**) RA value, (**b**) RMS, (**c**) kurtosis and (**d**) crest factor versus fatigue cycles of specimen No. 1. The change in crack growth rate is also included for understanding the AE behaviors in different stages.

Figure 4 and Figure 5 show the variations of eight time domain AE parameters as a function of fatigue cycles of specimen No. 1. The evolution of crack growth rate is also included for understanding the AE behaviors in different stages of FCG. Based on the results of different AE parameters and crack growth rates, one could see four obvious stages during FCG of the CrMoV steel. The four stages (i.e., stage A, B, C and D) are discriminated by black dashed lines in Figure 4 and Figure 5.

In stage A, noticeably high crack growth rates are observed. This is because after the onset of fatigue loading, the specimen undergoes significant plastic deformation and the crack starts to initiate from the notch. At the same time, a number of AE signals emerge in this stage. A large proportion of signals show low amplitudes of less than 60 dB and counts of less than 50. However, some AE signals with higher amplitude and energy can also be sporadically observed. This is more evident in the plots of entropy and RA value because many data points with high numerical values can be easily seen, indicating the AE source is active in this stage.

When the crack propagates into stage B, however, the crack growth rate slightly decreases and remains low. The crack grows almost linearly and the crack size at the end of stage B is less than 2.7 mm, indicating this stage is related to stable crack growth with a low da/dN. In comparison with AE characteristics in stage A, one could see a decrease in the numerical value of each AE parameter in stage B. For instance, the majority of AE energy in stage B is less than 10 and the maximum is 20.44, which is greatly smaller than that in stage A. The entropy shows a more distinct downward trend since the maximum entropy decreases from 7.38 in stage A to 6.17 in stage B. Other AE parameters also show similar but not particularly obvious phenomena. The low AE activity and intensity coincide well with the low crack growth rate in the stable crack growth range.

During stage C, the crack length increases by approximately 5 mm, and the crack growth rate exhibits an obvious increase and significant fluctuations compared with that in stage B. This evidence suggests stage C corresponds to the stable crack growth with a higher da/dN. In comparison with AE characteristics in stage B, all AE parameters show a distinct sudden rise. The numerical values of AE parameters of the majority of signals are much higher than those in stage B, indicating an enhanced AE activity.

When the crack further grows into stage D, which is close to the final failure of the specimen, a substantial rise in crack growth rate along with dramatic fluctuations can be evidently observed. The crack size increases by more than 12 mm and the crack growth rate increases about six times. These observations indicate stage D is associated with rapid or unstable crack growth before failure. Similar to the AE characteristics in stage C, the numerical values of all AE parameters in stage D exhibit a substantial increase. For instance, the maximum values of count and kurtosis are almost double those in stage C. Such distinct AE results are in good agreement with the rapid increase of da/dN in the unstable crack growth stage.

**Figure 6:**
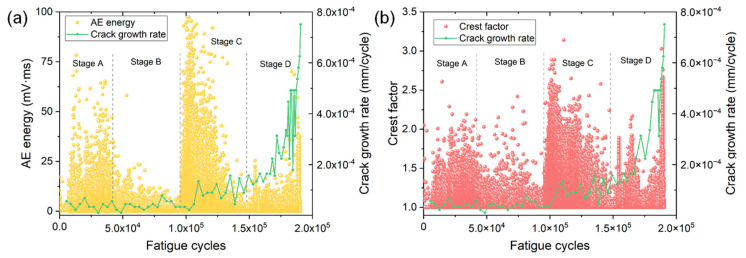
The variations of (**a**) AE energy and (**b**) crest factor versus fatigue cycles of specimen No. 2.

Figure 6 shows the variations of AE energy and crest factor as a function of fatigue cycles of specimen No. 2. Similar to specimen No. 1, four stages of FCG can be easily identified from the variations of AE parameters. The stage B shows the lowest AE intensity with a low da/dN, while stages C and D exhibit much higher AE energy and crest factor than those in stages A and B. At the end of stage D, a sudden increase in AE energy and crest factor can be observed due to the rapid increase in da/dN and final fracture.

**Figure 7:**
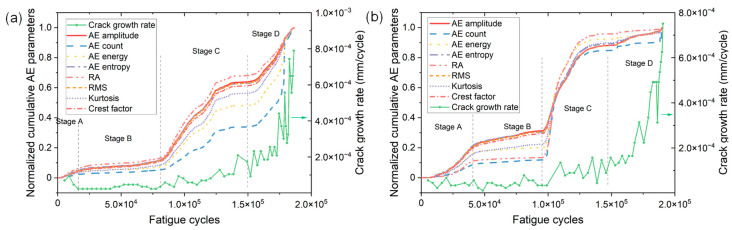
The variations of normalized cumulative AE parameters versus fatigue cycles of (**a**) specimen No. 1 and (**b**) specimen No. 2.

Figure 7a,b show the variations of normalized cumulative parameters as a function of fatigue cycles of specimen No. 1 and No. 2, respectively. The variation of crack growth rate is also shown for correlating different AE parameters. All the AE parameters exhibit a similar growth behavior during the fatigue process of two specimens. The cumulative AE parameters increase slowly in the first half of the fatigue life (i.e., stages A and B). However, a rapid increase in each cumulative parameter emerges in stage C where the crack propagates with a higher da/dN. This phenomenon is more obvious in specimen No. 2. Therefore, the rapid increase in AE in stage C can provide early and effective detection of accelerated crack growth, which is earlier compared to the warning sign provided by the crack growth rate. Despite the growth rate of AE parameters gradually decreases at the later part of stage C, the cumulative parameters exhibit a sudden rapid rise near the end of stage D again due to the occurrence of final fracture. This phenomenon is more obvious from the variation of cumulative AE count, i.e., the blue dashed line.

**Figure 8:**
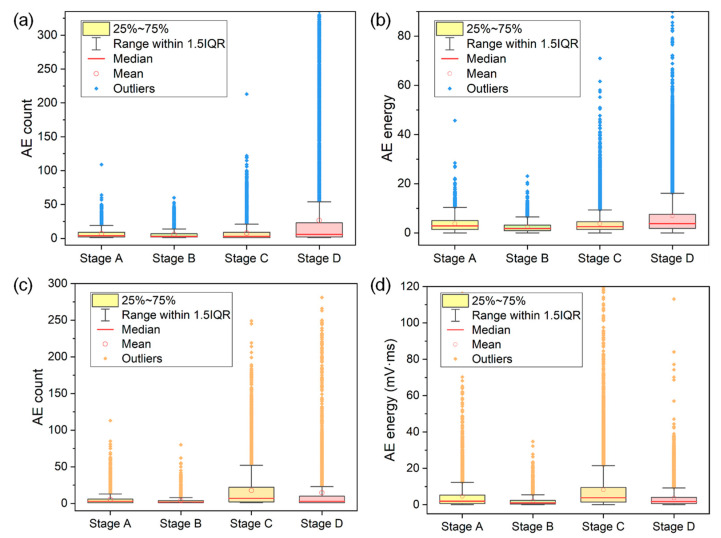
The distributions of (**a**,**c**) AE count and (**b**,**d**) energy in four stages of FCG of specimen (**a**,**b**) No. 1 and (**c**,**d**) No. 2. The colored regions represent the middle 50% of values, that is, the range between the 25% and 75% percentile. The range between the top and bottom lines is 1.5 times the interquartile range (IQR).

To further compare the AE characteristics during different stages of FCG, the AE count and energy of signals generated during each stage of two specimens were statistically analyzed and the results are illustrated in boxplots. As can be seen in Figure 8, in stages C and D, the AE count and energy greatly increase, and the ranges within 1.5IQR also significantly increase. Moreover, a number of outliers that are almost two times larger than those during the first two stages are observed. This is attributed to the increase in the activity and intensity of crack signals caused by accelerated crack growth rate in stages C and D. Therefore, the continuous emergence of a large number of AE signals with high count (>100) and high energy (>40 mV·ms) in stages C and D can help to provide early information for accelerated crack growth. Additionally, it is also important to note that both the range within 1.5IQR and the number of outliers in stage B are the minimum for both specimens, which coincides well with the low crack growth rate in stage B.

#### 3.2.2. AE Frequency Domain Parameter

**Figure 9:**
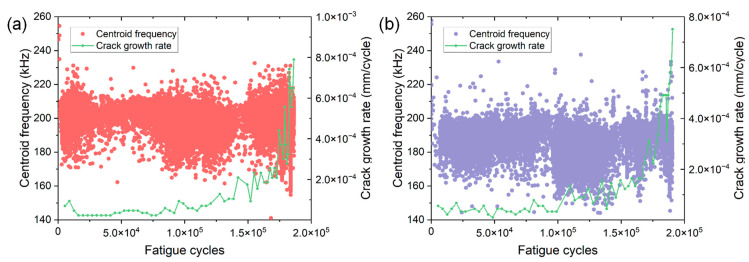
The variation of centroid frequency versus fatigue cycles of specimen (**a**) No. 1 and (**b**) No. 2.

Figure 9 shows the variation of centroid frequency as a function of fatigue cycles of two specimens. As the fatigue crack propagates, the centroid frequency does not seem to change significantly. Instead, the centroid frequency is mainly distributed in a narrow and stable range of 170–220 kHz for both specimens. Such a frequency band is in good agreement with the previous frequency results of AE signals generated from the high-frequency fatigue crack propagation of 316LN stainless steel [18]. Some recent studies have claimed that the significant change in frequency of AE signals can be regarded as the shift in failure mode inside the materials [24,27,28]. For instance, during the uniaxial tension of fiber metal laminate, it was found the frequency band of [0–100 kHz], [100–200 kHz], [200–300 kHz], and [300–400 kHz] of AE signals represent the matrix/metal crack, delamination, fiber pull-out and breakage, respectively [27]. Note that the AE sensor used in this study has an operating frequency range of [50–400 kHz] and can detect AE signals with a wide frequency range. The obtained narrow frequency band of [170–220 kHz] indicates the AE signals are mainly produced by crack growth rather than other sources during FCG. Compared with the time domain parameters used in this work, the frequency parameter may not be appropriate for characterizing different damage conditions during FCG because of the unobvious change in frequency. However, it is still recommended to analyze the frequency of AE signal because it helps to recognize different failure modes within the material. The occurrence of such frequency band during AE monitoring can provide evidence for possible crack growth within metallic materials.

#### 3.2.3. Coefficient of Variance of AE Data

To further quantify the performance of FCG characterization by different AE time domain parameters, the coefficient of variance (CV) of each parameter was calculated and compared. A CV measures relative dispersion of a dataset and is calculated by dividing the standard deviation by the mean [29]. A higher value of CV is generally favorable because it means a larger data dispersion, which is conducive to accurate damage identification. Table 2 presents the computed CV values of different AE parameters of both specimens. For specimen No. 1, the AE count shows the maximum value of CV, indicating the largest dispersion in count data. This result indicates that the count is the most proper AE parameter for qualitatively characterizing different stages during the FCG of specimen No. 1, which can be also proved by the excellent condition identification result in Figure 4b. It is also worth noting that the energy and RA exhibit significantly larger values of CV than other parameters. Therefore, the use of these two parameters can also provide complementary information for damage characterization. For specimen No. 2, the RA shows a maximum CV, followed by energy and count, which is similar to the specimen No. 1. In addition, the CV of amplitude is the lowest value among all AE parameters for both specimens. This reminds us that the only use of amplitude may lead to a large error in damage assessment.

In short, based on the combined analyses of AE multi-parameters and crack growth behavior, four stages of FCG can be distinctly identified and differentiated. Ostash has claimed that the fatigue fracture of materials generally includes three stages, that is, macrocrack initiation, macrocrack growth and spontaneous fracture [30]. The period of crack initiation and the period of subcritical growth of the macrocrack determine the fatigue lifetime of materials. Once the fatigue crack grows to a critical size, or the stress intensity factor reaches the cyclic fracture toughness of material, an unstable fracture occurs. In this study, stage A mainly corresponds to the macrocrack initiation, which includes the formation of the microcrack at the notch and its transformation into the macrocrack. Stages B, C and D are mainly associated with the growth of macrocrack with different crack growth rate. At the end of stage D, the rapid increase in AE is caused by unstable crack growth and fracture. The sudden and rapid increase in AE in stages C and D can provide important and effective warning signs for accelerated crack growth. This AE phenomenon is much earlier than the rapid increase of crack growth rate at the latter half of stage D, indicating the effectiveness of AE technique in FCG characterization. In addition, despite the frequency parameter of AE signals is not suitable for assessing the crack growth severity, it remains worthwhile to analyze because of its ability to distinguish different failure types within the material.

### 3.3. Quantitative Correlations between Crack Growth Rate and AE Parameters

**Figure 10:**
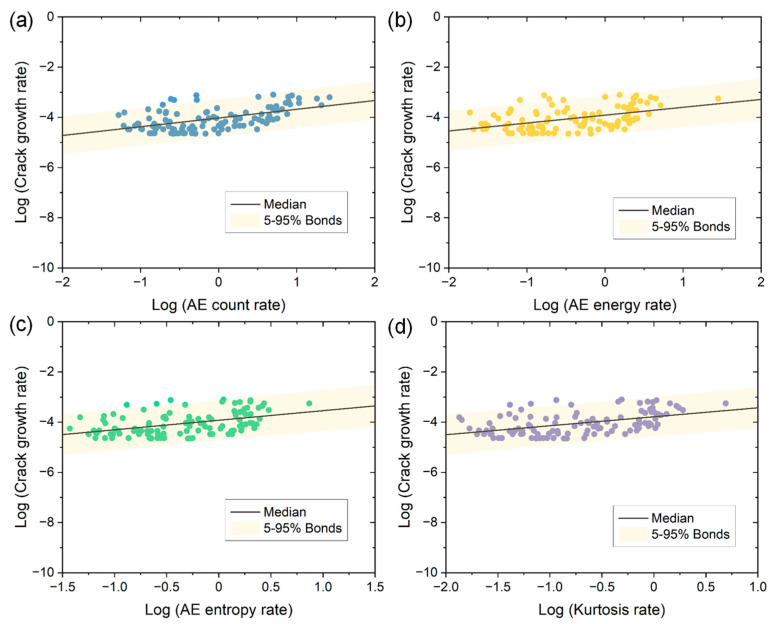
The logarithm of crack growth rate versus the logarithm of (**a**) AE count rate, (**b**) energy rate, (**c**) entropy rate, and (**d**) kurtosis rate of two specimens. The linear regression results including the median and the 95% prediction interval are also included.

Figure 10 presents the relationships between the logarithm of crack growth rate and the logarithms of AE time domain parameters involving the count rate, energy rate, entropy rate and kurtosis rate of two specimens in the linear coordinates. From Figure 10, an approximate linear correlation can be seen for all conditions despite the large data dispersion. This linear correlation can be expressed as follows:

(5) log(da/dN)=αlogX+β

where *α* and *β* are constants and can be determined by experiments, and *X* indicates the growth rate of AE data within a certain number of fatigue cycles *N*. Specifically, in this study, *X* can represent the AE amplitude rate, count rate, energy rate, entropy rate, RA rate, RMS rate, kurtosis rate and crest factor rate. The Equation (5) obtained in this work is also consistent with the previous results that there is an empirical linear relationship between crack growth rate and AE growth rate in long fatigue crack growth of metallic materials [14,15,16,18]. An important benefit of such correlation is that, once the AE monitoring data within a given range of fatigue cycles are obtained, the crack growth rate and crack size increase within such fatigue cycles can be predicted, which promotes the integrity assessment of engineering structures.

**Figure 11:**
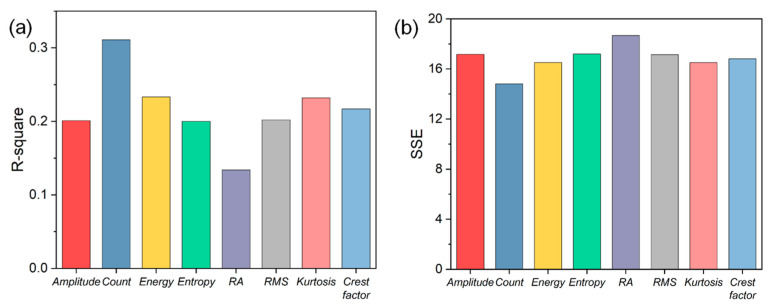
Linear regression results between crack growth rate and different AE parameters including the results of (**a**) R-square and (**b**) the sum of squares due to error (SSE).

Note that the accuracy of crack growth rate predictions depends on the use of the AE parameter in the quantitative relationship. If an improper AE parameter is used for constructing the linear correlation, a large error in the predicted crack growth rate may be obtained. To determine the superior AE parameters in describing Equation (5), linear least squares regressions are conducted. As shown in Figure 10, the median describes the best fit, and the yellow region denotes the 95% prediction interval where the majority of data are located. Furthermore, two important fitting goodness indicators including the R-square and the sum of squares due to error (SSE) are calculated and compared for different AE parameters, as shown in Figure 11. It can be seen that the best fitting regression result is achieved by AE count due to its highest R-square and the lowest SSE, indicating it is most appropriate for quantitatively correlating crack growth rate and AE data. Moreover, note that the count shows a very high CV for both parameters, as shown in Table 2. Thus, these results indicate AE count is the most effective parameter for qualitatively and quantitatively characterizing the FCG in CrMoV steel. From Figure 11 it can be also noted that the energy and kurtosis show significantly higher R-square and lower SSE compared with other AE parameters. At the same time, both of them have significantly higher values of CV than those of other parameters. Consequently, these two parameters (i.e., energy and kurtosis) can also serve as important candidates in describing FCG with small errors. Besides, linear regressions of amplitude, RMS, entropy and crest factor show little difference in performance, but are significantly worse than those based on count, energy or kurtosis. The RA value, however, shows the poorest linear regression performance due to the lowest R-square and highest SSE. Therefore, the RA value is not recommended as a qualified parameter for characterizing FCG in this study despite the fact that it exhibits a high CV for both specimens.

### 3.4. Fatigue Fracture Mechanism

**Figure 12:**
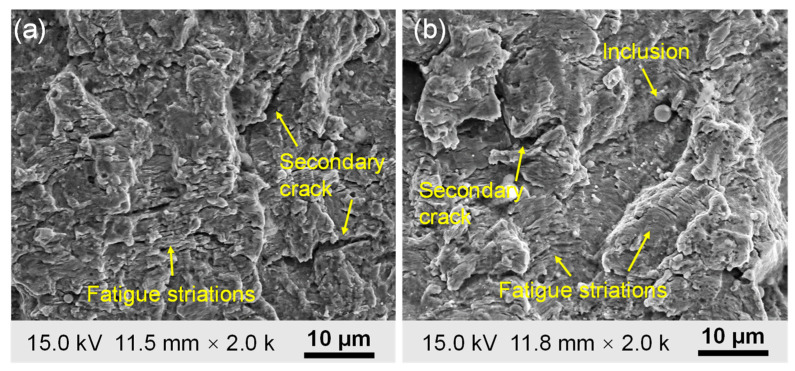
Typical fatigue-fracture surface morphology observed at the crack length of (**a**) 5.1 mm (Δ*K* = 22.94 MPa·m^1/2^, da/dN = 6.96·10^−5^ mm/cycle) and (**b**) 16.6 mm (Δ*K* = 40.25 MPa·m^1/2^, da/dN = 2.78·10 ^−4^ mm/cycle). The direction of fatigue crack propagation is from bottom to top.

The tested specimen shows a typical brittle fracture with a flat fracture surface. To further understand the microcosmic mechanism of FCG, the fracture surface morphology of 2.25Cr1Mo0.25V steel was investigated with the aid of SEM. Figure 12a,b show typical fatigue-fracture surface morphology observed at the crack size of 5.1 and 16.6 mm, respectively. It can be seen that the FCG of 2.25Cr1Mo0.25V steel is a transgranular fatigue fracture in nature. Numerous fatigue striations, which are reflective of fatigue crack propagation, can be obviously found on the fracture surface. The formation of fatigue striations is attributed to the blunting and sharpening of the crack tip under fatigue loading [31]. In addition to fatigue striations caused by the growth of the main crack, several secondary cracks can be observed on the fracture surface. Therefore, this evidence indicates the growth of the main crack and secondary cracks contributes to the generation of AE signals during fatigue loading.

## 4. Conclusions

In the present investigation, an AE multi-parameter analysis was performed for characterizing the FCG behavior of 2.25Cr1Mo0.25V steel. In particular, a variety of time domain parameters (i.e., amplitude, count, energy, information entropy, RA, RMS, kurtosis and crest factor) and frequency domain parameter (i.e., centroid frequency), were calculated for qualitatively assessing the crack growth condition and quantitatively correlating the crack growth rate with AE data. The performance of each AE parameter was analyzed and discussed. Major conclusions can be drawn as follows:

(1) Based on the combined analyses of AE time domain parameters and crack growth rate, four stages of FCG (i.e., stage A, B, C and D) of 2.25Cr1Mo0.25V steel can be distinguished. The four stages correspond to crack initiation, stable crack growth with low crack growth rate, stable crack growth with high crack growth rate, and unstable crack growth, respectively. The continuous emergence of a large number of AE signals with high count (>100) and high energy (>40 mV·ms) in stages C and D can help to provide early and effective warning signs for accelerated crack growth.
(2) The centroid frequency of AE signals caused by FCG of 2.25Cr1Mo0.25V steel is distributed in a narrow range of 170–220 kHz. The centroid frequency may not be appropriate for assessing the crack growth condition due to low variability, however, the occurrence of such a frequency band can help to identify possible crack growth signals.
(3) Linear correlations are found between crack growth rate and different AE parameters for quantifying crack growth. However, it should be noted that these quantitative correlations are only valid in current laboratory conditions. This is because AE signals are highly influenced by the sensor/source distance, specimen’s geometry and coupling quality [2], and consequently the quantitative relationships between AE and crack growth rate may not be obtained in the industrial environment. Before the practical application of this approach, the above-mentioned factors should be taken into account to reach a reliable quantification of fatigue crack of engineering structures.
(4) The AE multi-parameter analysis is recommended for damage characterization due to its advantage of reducing errors in using individual AE parameters. In this study, based on the multi-parameter analysis, one can conclude the count, energy and kurtosis are superior parameters for both qualitatively and quantitatively characterizing the FCG of 2.25Cr1Mo0.25V steel.

## Figures and Tables

**Table 1:** Definitions of different AE parameters used in the present research work.

AE Parameter	Definition
Amplitude/peak amplitude	Largest voltage peak of the signal waveform. It is expressed in a decibel scale where 1 μV at the sensor is defined as 0 dB.
Count/ring-down count	Number of times where AE signal exceeds the employed threshold
Energy	Measured area under the rectified signal envelope above the threshold
Information entropy	Information or Shannon’s entropy of AE waveform. It denotes the disorder or uncertainty of the probability amplitude distribution.
Rise time	Time interval between the point where the AE signal exceeds the threshold and the point where the peak amplitude occurs
Duration	Time interval from the point where the AE signal exceeds the threshold to the last point where it crosses the threshold
Rise angle (RA)	Ratio of rise time to amplitude
Root mean square (RMS)	Square root of average of squared value of the signal
Kurtosis	Measure of the “tailedness” of the AE signal
Crest factor	Ratio of the peak value to the RMS value
Centroid frequency	Weighted average of the frequency content calculated by performing fast Fourier transform

**Table 2:** Coefficients of Variance (CV) of different AE parameters.

AE Parameter	Amplitude	Count	Energy	Entropy	RA	RMS	Kurtosis	Crest Factor
Specimen 1	0.045	2.406	1.402	0.235	1.693	0.115	1.003	0.244
Specimen 2	0.829	1.683	1.735	1.189	2.082	1.369	1.291	1.342

## Data Availability

The data used to support the findings of this study are available from the corresponding author upon request.
